# Complete genome sequence of the broad host range *Acinetobacter baumannii* phage EAb13

**DOI:** 10.1128/MRA.00341-23

**Published:** 2023-08-21

**Authors:** Katie R. Margulieux, Jordan T. Bird, Richard T. Kevorkian, Damon W. Ellison, Mikeljon P. Nikolich, Nino Mzhavia, Andrey A. Filippov

**Affiliations:** 1 Wound Infections Department, Bacterial Diseases Branch, Walter Reed Army Institute of Research, Silver Spring, Maryland, USA; 2 Department of Biochemistry and Molecular Biology, University of Arkansas for Medical Sciences, Little Rock, Arkansas, USA; 3 Bacterial Diseases Branch, Walter Reed Army Institute of Research, Silver Spring, Maryland, USA; Katholieke Universiteit Leuven, Leuven, Belgium

**Keywords:** *Acinetobacter baumannii*, phage EAb13, broad host range, complete genome sequence, siphovirus, lytic phage, therapeutic candidate

## Abstract

We describe the genome of a lytic phage EAb13 isolated from sewage, with broad activity against multidrug-resistant *Acinetobacter baumannii*. EAb13 is an unclassified siphovirus. Its genome consists of 82,411 bp, with 40.15% GC content, 126 protein-coding sequences, 1 tRNA, and 2,177 bp-long direct terminal repeats.

## ANNOUNCEMENT

*Acinetobacter baumannii* causes a growing number of severe nosocomial infections linked to extensively drug-resistant (XDR) and pandrug-resistant (PDR) strains (1). With the rise of XDR and PDR infections, phages have been successfully used as alternative therapeutics for the compassionate treatment of humans (2, 3). A challenge for phage therapy against *A. baumannii* infections is a typical narrow activity of phages (3, 4). Here, we describe the genome of phage EAb13 with an unusually broad host range that was able to lyse 91/105 (86.7%) of diverse drug-resistant *A. baumannii* isolates (5).

The EAb13 phage was isolated in 2020 from sewage collected in Washington, DC, using an XDR respiratory isolate of *A. baumannii,* MRSN 423159, for phage enrichment. Single plaque isolation was performed three times to ensure purity, the phage was propagated in broth, and its DNA was extracted with the QIAamp DNA Mini Kit (Qiagen, Germantown, MD), according to the manufacturer’s protocol. A library was constructed using the KAPA HyperPlus Kit (Roche Diagnostics, Indianapolis, IN), and sequencing was performed on an Illumina MiSeq (Illumina, Inc., San Diego, CA) with the MiSeq Reagent Kit v3 (600 cycles, 300 bp reads). The quality of 101,029 paired-end reads was evaluated with FastQC 0.11.9 (6), and the reads were trimmed with Trimmomatic (7), v0.39. The genome was assembled using Unicycler (8), its termini were identified with PhageTerm (9), and phage lifestyle was predicted with BACPHLIP (10). Protein coding sequences (CDSs) were annotated using Pharokka pipeline (11 - 21). Amino acid sequence similarity searches were performed using default parameters in DIAMOND (22, 23).

**Fig 1:**
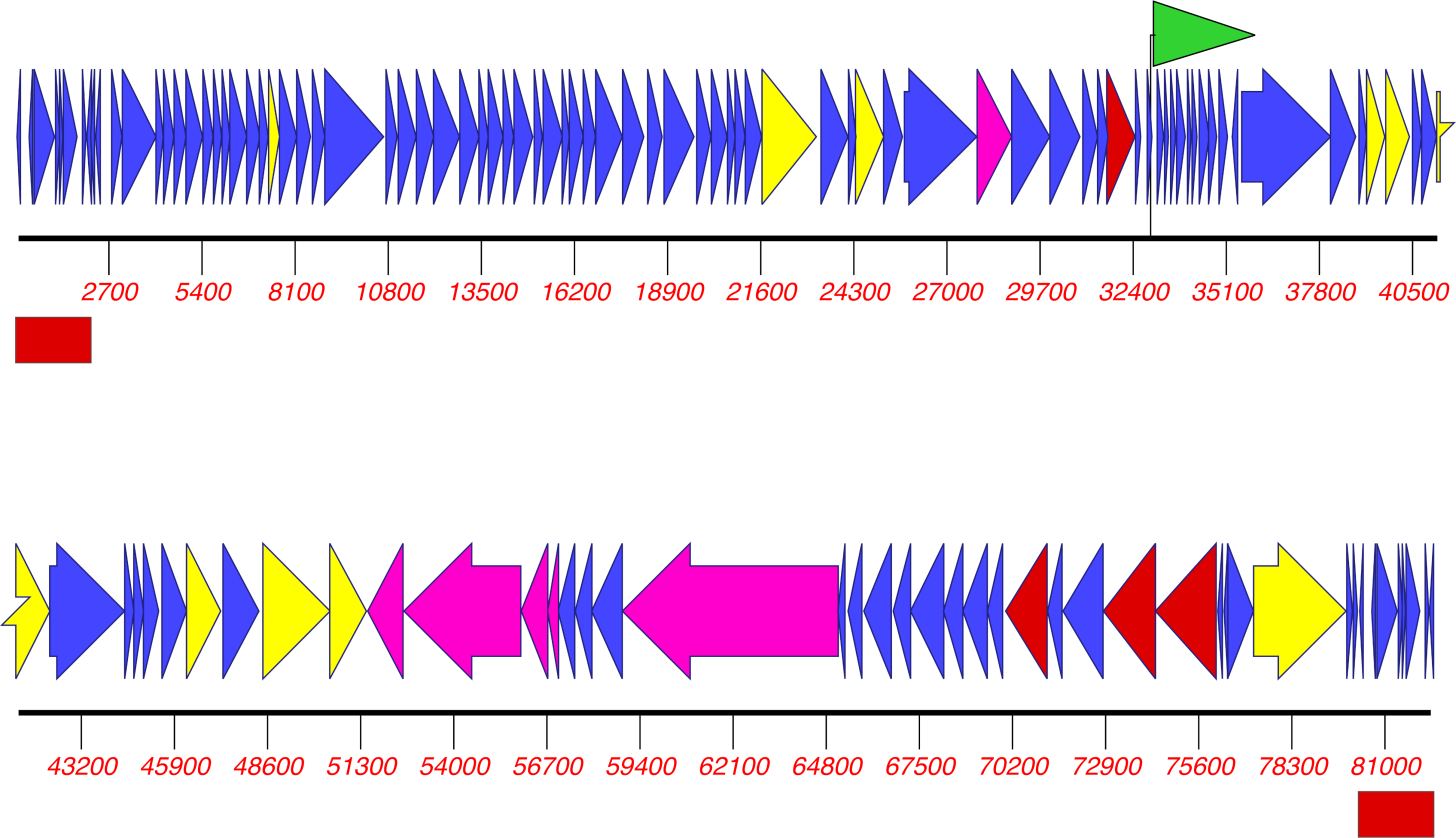
Genome organization of EAb13. Colored arrows denote the location of predicted coding sequences, transcriptional direction, and predicted function: unknown (blue), nucleotide metabolism (yellow), head and packing (red), and tail (pink). The green flag shows a tRNA sequence, and the red boxes mark terminal repeat regions.

The average read coverage was 430×; EAb13 genome length was 82,411 bp, with a G + C content of 40.15%, 126 predicted CDSs, and direct terminal repeats of 2,177 bp (Fig. 1). MASH analysis (21) against the INPHARED database (20) identified one phage with significant DNA identity to EAb13 (88.0%) across the entire genome, TCUP2199 (GenBank Accession Number ON323491; 24). Their terminase large subunit sequences shared 95.4% amino acid and 88.02% nucleotide identity (22). Of the 91 proteins that matched sequences in the nr database, 89 had top hits within TCUP2199. TCUP2199 was similar to EAb13 in genome length and GC content, also showed a broad host range, and has a siphovirus morphology based on electron microscopy (EM) (24). EM found that EAb13 has a long, non-contractile tail (data not shown), suggesting that this is also an unclassified phage with a siphovirus morphology that belongs to the class *Caudoviricetes*.

In contrast to TCUP2199, EAb13 encodes one tRNA, tRNA-Asn. Both phages encode several genes related to DNA synthesis (Fig. 1). The high (95.2%) BACPHLIP score suggested a virulent lifestyle of EAb13 (10). Its putative proteins did not show significant similarities to drug resistance or virulence determinants in the databases CARD (16) and VFDB (17), any other bacterial proteins, or phage products responsible for lysogenic lifestyle or gene transfer. Therefore, EAb13 appears to be a lytic phage and a promising candidate for therapeutic applications.

## Data Availability

The EAb13 genome BioProject, BioSample, GenBank, and NCBI Sequence Read Archive accession numbers are PRJNA948145, SAMN33875313, OQ717042, and SRX19782736, respectively.
